# Comparison of diagnostic performance of different imaging modalities for TAVI-patients

**DOI:** 10.1186/1532-429X-14-S1-P97

**Published:** 2012-02-01

**Authors:** Yang Chul Boering, Mirja Neizel, Florian Bönner, Sebastian Grünig, Marc W Merx, Tobias Zeus, Stefan Steiner, Jan Balzer, Malte Kelm, Burkhard Sievers

**Affiliations:** 1 Cardiology, Heinrich Heine University, Duesseldorf, Germany

## Background

In contrast to surgical aortic valve replacement pre procedural assessment of aortic, aortic annulus diameter and prosthesis size is essential for successful percutaneous interventional valve therapy. Among different imaging modalities multislice-computed tomography (MSCT) and transesophageal echocardiography (TEE)are routinely used for patient screening. We sought to evaluate the diagnostic performance of cardiac magnetic resonance tomography (CMR) in this setting, and the difference in diameter measurements between all 3 imaging modalities.

## Methods

30 patients (mean age 80±8 years, 19 male) were underwent TEE (Philips iE33, Andover, USA), MSCT (64-slice Siemens Somatom, Forchheim, Germany), and CMR (Philips Achieva, Best, Netherlands) studies to assess enddiastolic diameters of aortic annulus, aortic bulbus and ascending aorta. In addition, minimal aortic valve area was assessed by TEE and CMR. CT data were retrospectively triggered and reconstruction was set to 60-75% of the cardiac phase. CMR-studies were prospectively triggered using a free breathing navigator for acquisition of a full 3D volume data set (cardiac phase 50-80%). Subsequent image analysis for MSCT and CMR was performed using the vendor specific dedicated 3D analysis tools. TEE measurements were performed on 2D images of 3-chamber views.

## Results

Aortic annulus diameter was 24.2±2.8mm for MSCT, 23.36±2.4mm for CMR and 22.6±2.01mm for TEE (p<0.01). Aortic bulbus was measured at 33.71±3.6mm on MSCT, 32.75±2.4mm on CMR and 30.5±2.01mm on TEE images (p<0.01). Ascending aortic diameter was 30.63±5.04mm on MSCT, 28.43±4.11mm on CMR, and 28.5±4.08mm on TEE (p<0.01). Differences between measurements and imaging modalities were assessed by Bland Altman statistics. Mean SD for 1) aortic annulus measurements was 0.8±4.6mm for TEE versus CMR and 1.1±3.7 mm for MSCT versus CMR, 2) aortic bulbus diameters 2.2±2.7 mm for TEE versus CMR and 1.0±3.7 mm for MSCT versus CMR, and 3) ascending aorta 0.9±3.1mm for TEE versus CMR and 2.2±3.5 mm for MSCT versus CMR. The minimal aortic valve area showed good agreement between CMR and TEE, standard deviation 0.04±0.41 sqcm.

## Conclusions

Aortic diameter measurements based on MSCT images were significantly higher compared to CMR and TEE. The lowest diameters were consistently measured on TEE. Our findings may have a relevant clinical impact for decision making in pre procedural TAVI planning and work flow regarding size and type of aortic valve prosthesis.

## Funding

No funding.

